# National high prevalence, and low awareness, treatment and control of dyslipidaemia among people aged 15–69 years in Mongolia in 2019

**DOI:** 10.1038/s41598-022-14729-2

**Published:** 2022-06-21

**Authors:** Supa Pengpid, Karl Peltzer

**Affiliations:** 1 Department of Health Education and Behavioral Sciences, Faculty of Public Health, Mahidol University, Bangkok, Thailand; 2 Department of Psychology, University of the Free State, Bloemfontein, South Africa; 3 Department of Psychology, College of Medical and Health Sciences, Asia University, Taichung, Taiwan

**Keywords:** Diseases, Risk factors

## Abstract

The aim of the study was to evaluate the prevalence, distribution and correlates of dyslipidaemia among people (15–69 years) in Mongolia. National data were analyzed from 4,895 individuals (15–69 years, median age = 35 years) that took part in the Mongolia cross-sectional STEPS survey in 2019, and had complete lipid measurements. Dyslipidaemia was defined using the guidelines of the Adult Treatment Panel III. The prevalence of dyslipidaemia was 58.6%, 31.7% high triglycerides (TG), 26.9% high low-density lipoprotein cholesterol (LDL-C), 26.9% high total cholesterol (TC) and 14.6% low high-density lipoprotein cholesterol (HDL-C). Among those with dyslipidaemia, 6.2% were aware. Among those who were aware, the proportion of lipid-lowering drug treatment was 18.9% and among those who took lipid-lowering drugs, 21.5% had their dyslipidaemia controlled. In adjusted logistic regression, older age (40–69 years) (AOR: 1.19, 95% CI 1.02–1.40), urban residence (AOR: 1.24, 95% CI 1.04–1.48), obesity call II (AOR: 2.89, 95% CI 2.29–3.66), hypertension (AOR: 1.33, 95% CI 1.11–1.59), and diabetes (AOR: 1.62, 95% CI 1.20–2.18) were positively, and male sex (AOR: 0.84, 95% CI 0.72–1.00) was negatively associated with dyslipidaemia prevalence. Six in ten Mongolians 15 years and older had dyslipidaemia. Several factors associated with dyslipidaemia that can be used to target public health interventions were identified.

## Introduction

Dyslipidaemia constitutes one or a combination of elevated triglycerides (TG), high low-density lipoprotein cholesterol (LDL-C), low high-density lipoprotein cholesterol (HDL-C), and elevated total cholesterol (TC)^1,2^. Dyslipidaemias are associated with changes in the plasma lipid profile leading to major clinical conditions, such as cardiovascular disease (CVD), and the global burden has increased significantly over the past 30 years^1^. CVDs contribute to 40% of mortality in 2016 in Mongolia, which is a lower resource country located in East Asia^3^. Ischemic heart disease (IHD) is high and increasing in Mongolia, due to risk factors, such as dyslipidaemia, obesity, diabetes, smoking, and hypertension^4^. “Dyslipidaemias can be genetically determined (primary or familial dyslipidaemias) or secondary to other conditions (such as diabetes mellitus, obesity, or an unhealthy lifestyle), the latter being more common^1^.” Based on our review, we could not find any national data on dyslipidaemia in Mongolia.

In 35 low- and middle-income countries (LMIC) (≥ 15 years), the prevalence of high TC was 7.1% and high LDL-C 7.5%, ≥ 31% of them were aware of their diagnosis, ≥ 29% were treated, and ≥ 7% were controlled^5^. In China (> 18 years), the prevalence of dyslipidaemia was 34.0%^6^, and 31.0% were aware, 19.5% in treatment and 8.9% were controlled^6^. In northern Ethiopia (≥ 20 years), the prevalence of dyslipidaemia was 66.7%, elevated TC 30.8%, elevated TG 40.2%, low HDL-C 16.5%, and high LDL-C 49.5%^2^, in Jordan (≥ 25 years) the prevalence of elevated TC was 44.3%, high TC 41.9%, and low HDL-C 59.5%^7^, in Turkey (≥ 20 years), high LDL-C was 36.2%, high TG 35.7%, low HDL-C 41.5%, and high TC was 43%, and dyslipidaemia (at least one abnormal lipid value) ≥ 79%^8^, and in Pakistan (≥ 20 years) high TC 39.3%, high TG 48.9%, high LDL-C 39.7%^9^.

Sociodemographic factors associated with dyslipidaemia include increased age^2,6^, male sex^6,10^, ethnicity^10^, and living in urban areas^6,10^. Health risk factors associated with dyslipidaemia include obesity^2,6,8,10,11^, central obesity^10^, cardiovascular disease^6^. diabetes^6,8,10–12^, hypertension^6,8,10–12^. Health behaviour risk factors associated with dyslipidaemia, include smoking^10^, physical inactivity^2^, exposure to secondary smoke^13^, and alcohol use had lower odds of dyslipidaemia^10^.

Factors associated with high TC include older age^14^, female sex^14^, lower education^9,14,15^, hypertension^7,9,12^, diabetes^7,9,12,14^, obesity^7,9^, and smoking^9^. Factors associated with high LDL-C include increasing age^14^, female sex, hypertension, diabetes, and obesity^9^. Factors associated with low HDL-C include, decreasing age^14^, female sex^9^, male sex^14^, higher education^14^, hypertension^7^, diabetes^7,9,14^, and obesity^7,9,14^ cigarette smoking^14,16^. Factors associated with high TG include male sex^12,14^, higher education^14^, hypertension^7,12,14^, diabetes^7,9,12,14^, obesity^7,9,12^, physical inactivity^14^, and smoking or current tobacco use^7,12,14^.

Factors associated with awareness of dyslipidaemia diagnosis include older age^5^, living in urban areas^11^, higher education^11^, higher BMI^5,11,17^, comorbid diagnosis of diabetes^5,11^, hypertension^5,11^, and CVD^11^. Factors associated with the treatment of dyslipidaemia or hypercholesterolemia include women^7^, comorbid hypertension, diabetes^5^, and CVD^11^. Factors positively associated with control of dyslipidaemia include women^7^, urban residence^7^, obesity^7^, comorbid diabetes^5^, and CVD^11^, and overweight/obesity and physical inactivity were negatively associated with control of dyslipidaemia^11^. The aim of the study was to assess the prevalence, distribution, and correlates of dyslipidaemia among people aged 15 to 69 years in Mongolia.

## Methods

### Participants and procedures

Cross-sectional national data with complete lipid measurements from the 2019 Mongolia STEPS survey^18^ were analyzed; the study response rate was 98.1%^19^. According to STEPS survey procedures,

“Socio-demographic and behavioural information was collected in Step 1. Physical measurements such as height, weight, and blood pressure were collected in Step 2. Biochemical measurements were collected to assess blood glucose and cholesterol levels in Step 3. A multi-stage stratified sampling process (377 sampling units or clusters selected from 21 provinces and 9 districts of Ulaanbaatar) was carried out to randomly select participants from the target population. One individual within the age range of the survey (15–69 years) was selected per household. If the randomly selected individual had temporarily been out of the range of the survey clusters (soum/khoroo) during the whole period of the field work, he or she was excluded and re-sampling was conducted. Ethics approval was provided by the Ministry of Health Medical Ethical Committee, Mongolia, and written informed consent was obtained from all participants, including from a parent and/or legal guardian^18,19^.”

All methods were performed in accordance with the relevant guidelines and regulations.

Data collection followed the “WHO three STEPS methodology: step 1 included administration of a structured questionnaire (sociodemographics, medical history, medication use, and health risk behaviour) step 2 consisted of blood pressure and anthropometric measurements, and step 3 included biochemical tests (blood glucose and blood lipids)^18^.” Anthropometric measurements were taken using the “Somatometre-Stanley 04-116 device and electronic scales GIMA”)^19^. Of the three blood pressure measurements using “OMRON Model M5 automatic blood pressure monitor^19^,” the last two readings were averaged^18^. “Blood glucose, total cholesterol and triglycerides were measured in peripheral (capillary) blood at the data collection site using dry chemical methods using multi-functional ‘Prima home test’ diagnostic device, biochemical analysis and automated analyzer^19^.” Serum samples were taken to analyze LDL and HDL cholesterol^19^. Laboratory analysis included blood glucose, TC, TG, HDL, and LDL. Laboratory tests for LDL and HDL in blood, were performed and analyzed in “Gyals” LLC’s laboratory using biochemical automated analyzer. HDL and LDL content was measured in serum using a direct or two-point linear method^19^.

### Measures

Dyslipidaemia was classified^20^ as:

“being on antilipidemic medication or having one or more of the following: elevated total cholesterol (TC): ≥ 5.17 mmol/l (200 mg/dl), high triglycerides (TG): ≥ 1.70 mmol/l (150 mg/dl), low HDL-C: female ≤ 1.29 mmol/l; male ≤ 1.03 mmol/l (50 mg/dl in women, 40 mg/dl in men) and high LDL-C: ≥3.36 mmol/l (130 mg/dl).”

The awareness rate of dyslipidaemia was defined as:

“having been diagnosed by a health care provider as having high cholesterol among those with dyslipidaemia. The rate of dyslipidaemia treatment was defined as the self-reported use of lipid lowering drugs among participants who were aware of dyslipidaemia. The control rate of dyslipidaemia was classified as the proportion among those treated for dyslipidaemia who reach the lipid standard: TG < 1.70 mmol/L, TC < 5.18 mmol/L, HDL-C ≥ 1.04 mmol/L and LDL-C < 3.37 mmol/L^20^.”

*Other biological measures* included measured central obesity (“waist circumference ≥ 90 cm in men, ≥ 80 cm in women”)^21^; measured *Body Mass Index* (BMI) (kg/m^2^): “normal weight 18.5–22.9 kg/m^2^, underweight < 18.5 kg/m^2^, overweight 23.0–24.9 kg/m^2^, obesity class I 24.9 kg/m^2^, − 29.9 kg/m^2^, and obesity class II ≥ 30.0 kg/m^2^^22^”, *Hypertension/raised blood pressure* (BP): “systolic BP ≥ 140 mm Hg and/or diastolic BP ≥ 90 mm Hg or where the participant is currently on antihypertensive medication^23^.” *Diabetes:* “fasting plasma glucose levels ≥ 7.0 mmol/L (126 mg/dl); or using insulin or oral hypoglycaemic drugs; or having a history of diagnosis of diabetes^24^.”

*Behavioural measures* included current tobacco use, current heavy episodic drinking (“six or more standard drinks in a single drinking occasion”), exposure to secondary smoke, daily consumption of fruits and vegetables, and low, moderate and high physical activity and sedentary behaviour (≥ 8 h/day) based on the “Global Physical Activity Questionnaire^25^.” Sociodemographic variables included age (years), sex (male, female), education in years, region, residence status, and ethnic group^19^.

History of CVDs included self-reported “Have you ever had a heart attack or chest pain from heart disease (angina) or a stroke (cerebrovascular accident or incident)? (Yes, No)^19^.”

### Data analysis

All statistical analyses were conducted with “STATA software version 14.0 (Stata Corporation, College Station, TX, USA).” “Analysis weights were calculated by taking the inverse of the probability of selection of each participant. These weights were adjusted for differences in the age-sex composition of the sample population as compared to the target population^19^.” Descriptive statistics are used to describe lipid profiles. Multivariable logistic regressions were used to assess the associations between sociodemographic (age, gender, education, and residence status) and health factors (BMI, hypertension, diabetes, cardiovascular disease, physical activity, sedentary behaviour, fruit/vegetable intake, current tobacco use, passive smoking, and heavy episodic drinking) and dyslipidaemia profiles as well as awareness and treatment. Afterward, significant variables in unadjusted analyses were included in the multivariate model. Covariates were selected based on previous literature review^2,6,8,10–17^. To account for the multi-stage sample design, Taylor linearization methods were utilized. P-values < 0.05 were considered significant. Statistical analyses were computed with complete cases of lipid profile measurement. Of the 6654 total study sample, 4895 had complete measures of lipid profile. The 1759 excluded individuals with an incomplete lipid profile were more likely to be younger, but did not differ in terms of residence status, sex, educational level, and ethnicity. Furthermore, sensitivity testing was done using multiple imputation by chained equations to fully impute the dataset. The logistic regression with dyslipidaemia as outcome was conducted with complete imputed data and results were compared with complete case analyses results.

## Results

### Sample characteristics

The sample with complete lipid measurements included 4,895 persons (15–69 years), with a median age of 35 years (IQR: 25–47 years) in 2019.

The prevalence of dyslipidaemia was 58.6% (54.5% using the restricted lipid profile, including high TC, high TG, and low HDL-C), 26.9% high TC, 31.7% high TG, 26.9% high LDL-C and 14.6% low HDL-C. Among the five regions in Mongolia, the highest proportion of dyslipidaemia was found in the central region (63.4%) and Ulaanbaatar (61.8%) and the lowest in the western region (48.8%). Further sample characteristics are described in Table 1 (see Table 1).

**Table 1:** Sample description and prevalence of dyslipidaemia and its subtypes, Mongolia 2019.

Variable	Subcategory	Sample	Dyslipidaemia	High TC	High TG	Low HDL-C	High LDL-C
		N (%)	%	%	%	%	%
Total		4895	58.6	26.9	31.7	14.6	26.9
Age (years)	15–24	477 (22.3)	48.4	14.4	23.0	7.2	27.5
	25–34	1091 (25.4)	56.6	23.4	29.3	12.3	27.3
	35–44	1181 (21.2)	62.4	29.0	36.1	17.1	27.1
	45–54	1026 (18.4)	63.5	35.9	36.5	18.5	24.9
	55–69	1120 (12.6)	67.3	39.3	37.7	22.4	27.2
Gender	Female	2661 (49.2)	60.5	24.7	30.5	12.9	32.0
	Male	2234 (50.8)	56.8	29.0	32.9	16.2	21.9
Education (in years)	0–9	1373 (24.7)	56.6	26.2	30.5	13.4	24.0
	10–11	1271 (25.7)	57.9	27.0	30.4	15.2	27.3
	≥ 12	2251 (49.6)	60.0	27.2	33.0	14.9	28.1
Ethnic group	Other	712 (14.8)	54.3	25.7	29.7	12.4	22.6
	Khalkh	4162 (85.2)	59.5	27.2	32.1	15.0	27.8
Residence	Rural	1686 (36.4)	55.0	25.0	29.5	12.0	24.3
	Urban	3209 (63.6)	60.7	27.9	33.0	16.1	28.4
Region	Central	697 (16.3)	63.4	36.7	37.2	18.9	22.5
	Eastern	494 (7.9)	54.1	17.9	27.8	9.6	29.9
	Khangai	876 (20.1)	55.7	23.9	27.6	12.0	28.7
	Ulaanbaatar	2216 (43.2)	61.8	26.3	33.1	16.1	31.2
	Western	612 (12.4)	48.8	26.4	28.7	11.1	12.6
Body Mass Index	Normal	1073 (28.1)	45.3	18.3	19.3	9.1	20.1
	Underweight	107 (3.3)	53.2	14.0	17.9	6.5	28.1
	Overweight	716 (15.6)	53.8	25.0	26.2	13.8	24.4
	Obesity class I	1703 (32.5)	62.3	30.3	36.0	16.9	27.2
	Obesity class II	1136 (20.6)	73.8	36.2	46.4	20.6	36.8
Central obesity	No	1703 (43.3)	48.7	19.5	21.7	10.3	22.2
	Yes	3070 (56.7)	65.6	32.3	38.8	18.0	30.4
Hypertension	No	3341 (74.7)	54.9	24.1	28.4	12.9	25.5
	Yes	1502 (25.3)	69.0	35.5	41.1	19.9	30.2
Diabetes	No	4244 (91.4)	57.0	25.9	29.4	14.5	26.2
	Yes	451 (8.6)	72.9	34.1	50.3	14.6	34.5
Cardiovascular disease	No	4071 (85.1)	58.5	26.8	31.6	14.3	26.9
	Yes	822 (14.9)	59.0	27.5	32.2	16.2	26.8
Physical activity	Low	1524 (30.2)	63.0	31.3	38.7	17.0	30.4
	Moderate	901 (18.2)	58.0	27.9	31.5	16.9	26.8
	High	2385 (51.6)	56.5	23.9	27.9	12.6	25.2
Sedentary	No	4223 (88.6)	57.9	26.3	31.4	14.2	26.8
	Yes	614 (11.4)	63.6	31.6	34.2	18.2	27.4
Fruit/vegetable intake	≥ 5 servings	934 (21.3)	60.7	23.6	33.2	12.6	31.7
	< 5 servings	3735 (78.7)	58.2	27.9	31.2	15.3	25.8
Current tobacco use	No	3646 (74.4)	58.2	25.4	30.2	13.8	27.2
	Yes	1249 (25.6)	59.7	31.1	36.1	17.1	25.8
Passive smoking	No	2769 (58.1)	57.9	27.1	32.3	14.8	25.6
	Yes	2026 (41.9)	59.7	26.6	31.0	14.4	28.6
Heavy drinking	No	3800 (79.6)	57.9	25.4	30.2	14.2	28.2
	Yes	1012 (20.4)	62.4	33.0	37.5	16.6	22.3

### Distribution of dyslipidaemia awareness, treatment, and control

Among those with dyslipidaemia, 6.2% were aware. Among those who knew, the proportion of lipid-lowering drug treatment was 18.9%, and among those taking lipid-lowering drugs, 21.5% had their dyslipidaemia controlled (see Table 2).

**Table 2:** Dyslipidaemia awareness, treatment, and control.

Variable	Subcategory	Dyslipidaemia		
		Awareness (N = 2972)	Treatment (N = 322)	Control (N = 65)
		%	%	%
Total		6.2	18.9	21.5
Age (years)	15–24	0.2	0.0	–
	25–34	5.4	18.0	0.0
	35–44	6.5	9.9	39.9
	45–54	10.2	22.6	20.5
	55–69	9.2	25.8	25.2
Gender	Female	7.7	16.8	22.2
	Male	4.7	22.7	19.4
Education (in years)	0–9	4.3	21.2	69.7
	10–11	5.0	14.5	43.7
	≥ 12	7.7	19.9	9.1
Ethnic group	Other	5.3	11.9	100.0
	Khalkh	6.3	20.0	14.4
Residence	Rural	4.0	18.9	20.8
	Urban	7.3	18.9	21.6
Region	Central	5.0	17.8	24.6
	Eastern	5.3	35.6	67.4
	Khangai	3.7	19.6	36.7
	Ulaanbaatar	8.5	18.0	12.7
	Western	3.3	15.6	–
Body Mass Index	Normal	1.9	23.8	100.0
	Underweight	0.5	0.0	–
	Overweight	5.1	12.4	47.4
	Obesity class I	6.5	17.3	45.6
	Obesity class II	11.1	21.1	0.0
Central obesity	No	2.5	17.7	47.3
	Yes	8.4	19.1	21.8
Hypertension	No	5.3	17.7	41.5
	Yes	8.5	19.9	12.5
Diabetes	No	5.6	17.0	21.4
	Yes	10.4	27.6	26.8
Cardiovascular disease	No	5.1	18.2	18.3
	Yes	12.3	21.1	28.3
Physical activity	Low	5.8	24.8	18.4
	Moderate	7.7	17.4	15.2
	High	5.9	16.7	49.4
Sedentary	No	4.7	17.5	12.3
	Yes	6.3	28.5	33.5
Fruit/vegetable intake	≥ 5 servings	7.0	20.3	6.2
	< 5 servings	6.1	17.8	38.5
Current tobacco use	No	6.4	15.6	41.8
	Yes	5.5	29.7	0.0
Passive smoking	No	5.6	16.1	17.7
	Yes	7.1	21.7	24.0
Heavy episodic drinking	No	6.7	18.3	23.3
	Yes	4.0	18.1	0.0

### Associations with dyslipidaemia prevalence

In adjusted logistic regression, older age (40–69 years) (AOR: 1.19, 95% CI 1.02–1.40), urban residence (AOR: 1.24, 95% CI 1.04–1.48), obesity call II (AOR: 2.89, 95% CI 2.29–3.66), hypertension (AOR: 1.33, 95% CI 1.11–1.59), and diabetes (AOR: 1.62, 95% CI 1.20–2.18) were positively and male sex (AOR: 0.84, 95% CI 0.72–1.00) was negatively associated with dyslipidaemia prevalence. Furthermore, in the unadjusted analysis, episodic heavy drinking was positively associated and increased physical activity was negatively associated with the prevalence of dyslipidaemia (see Table 3).

**Table 3:** Associations with prevalence of dyslipidaemia.

	Subcategory	COR (95% CI)	AOR (95% CI)^a^
Age (years)	15–39	1 (reference)	1 (reference)
	40–69	1.54 (1.35, 1.77)***	1.19 (1.02, 1.40)*
Gender	Female	1 (reference)	1 (reference)
	Male	0.86 (0.74, 1.00)*	0.84 (0.72, 1.00)*
Education (in years)	0–9	1 (reference)	–
	10–11	1.05 (0.86, 1.29)	
	≥ 12	1.15 (0.97, 1.36)	
Ethnic group	Other	1 (reference)	–
	Khalkh	1.23 (0.98, 1.55)	
Residence	Rural	1 (reference)	1 (reference)
	Urban	1.27 (1.07, 1.50)**	1.24 (1.04, 1.48)*
Body Mass Index	Normal	1 (reference)	1 (reference)
	Underweight	1.37 (0.84, 2.24)	1.29 (0.78, 2.13)
	Overweight	1.40 (1.12, 1.76)**	1.37 (1.08, 1.73)**
	Obesity class I	2.00 (1.65, 2.42)***	1.81 (1.48, 2.21)***
	Obesity class II	3.40 (2.72, 4.24)***	2.89 (2.29, 3.66)***
Hypertension	No	1 (reference)	1 (reference)
	Yes	1.67 (1.44, 1.93)***	1.33 (1.11, 1.59)**
Diabetes	No	1 (reference)	1 (reference)
	Yes	2.03 (1.55, 2.68)***	1.62 (1.20, 2.18)**
Cardiovascular disease	No	1 (reference)	–
	Yes	1.02 (0.84, 1.24)	
Physical activity	Low	1 (reference)	1 (reference)
	Moderate	0.81 (0.66, 1.00)*	0.94 (0.75, 1.18)
	High	0.76 (0.64, 0.90)***	0.94 (0.79, 1.12)
Sedentary	No	1 (reference)	–
	Yes	1.27 (1.03, 1.57)	
Fruit/vegetable intake	≥ 5 servings	1 (reference)	–
	< 5 servings	0.90 (0.75, 1.08)	
Current tobacco use	No	1 (reference)	–
	Yes	1.06 (0.91, 1.24)	
Passive smoking	No	1 (reference)	–
	Yes	1.08 (0.92, 1.26)	
Heavy episodic drinking	No	1 (reference)	1 (reference)
	Yes	1.21 (1.02, 1.43)*	1.19 (0.98, 1.44)

*COR* crude odds ratio, *AOR* adjusted odds ratio.

***p < 0.001; **p < 0.01; *p < 0.05.

^a^All variables significant (*p* < 0.05) in unadjusted analyses were included in the adjusted model.

Furthermore, testing the consistency of these results using multiple imputation of missing data in the dependent variable were explored. The direction and significance of the relationship between covariates and dyslipidaemia in the two models (complete cases: Table 3 and imputed model: Supplementary Table 1) were similar to except in sex, education, and heavy episodic drinking where the level of significance differed).

### Associations with prevalence of dyslipidaemia subcategories

In the adjusted logistic regression analysis, older age was positively associated with high TC, high TG, and low HDL-C, and inversely associated with high LDL-C. Male sex was positively associated with high TC and low HDL-C, and negatively associated with high LDL-C. Higher education was associated with high TC, high TG, and low LDL-C. Belonging to the Khalkh ethnic group was positively associated with high LDL-C. Obesity was associated with the four dyslipidaemia subcategories. Hypertension increased the odds of high TC and low LDL-C, and diabetes increased the odds of high TG and high LDL-C. High physical activity reduced the odds high TC, high TG, and low LDL-C. Current tobacco use was positively associated with high TC, high TG, and high LDL-C, while heavy alcohol use was positively associated with high TG (see Table 4).

**Table 4:** Associations with prevalence of dyslipidaemia subcategories.

Variable	Subcategory	High TC	High TG	Low HDL-C	High LDL-C
		AOR (95% CI)	AOR (95% CI)	AOR (95% CI)	AOR (95% CI)
Age (years)	15–39	1 (reference)	1 (reference)	1 (reference)	1 (reference)
	40–69	1.91 (1.61, 2.28)***	1.27 (1.08, 1.50)**	1.63 (1.29, 2.05)***	0.72 (0.61, 0.85)***
Gender	Female	1 (reference)	1 (reference)	1 (reference)	1 (reference)
	Male	1.38 (1.14, 1.67)***	1.11 (0.93, 1.32)	1.39 (1.08, 1.78)*	0.59 (0.48, 0.72)***
Education (in years)	0–9	1 (reference)	1 (reference)	1 (reference)	1 (reference)
	10–11	1.21 (0.97, 1.50)	1.24 (1.01, 1.52)*	1.25 (0.93, 1.66)	1.19 (0.96, 1.46)
	≥ 12	1.26 (1.03, 1.55)*	1.47 (1.20, 1.80)***	1.31 (1.01, 1.70)*	1.05 (0.86, 1.28)
Ethnic group	Other	1 (reference)	1 (reference)	1 (reference)	1 (reference)
	Khalkh	1.02 (0.81, 1.29)	1.16 (0.93, 1.48)	1.17 (0.85, 1.62)	1.45 (1.08, 1.93)*
Residence	Rural	1 (reference)	1 (reference)	1 (reference)	1 (reference)
	Urban	1.02 (0.83, 1.27)	1.05 (0.87, 1.28)	1.31 (1.00, 1.73)	1.08 (0.89, 1.32)
Body Mass Index	Normal	1 (reference)	1 (reference)	1 (reference)	1 (reference)
	Underweight	0.57 (0.28, 1.18)	0.86 (0.48, 1.53)	0.97 (0.43, 2.20)	1.19 (0.75, 1.88)
	Overweight	1.63 (1.24, 2.14)***	1.50 (1.14, 1.98)**	1.64 (1.14, 2.37)**	1.16 (0.87, 1.55)
	Obesity class I	1.99 (1.58, 2.51)***	2.34 (1.90, 2.88)***	1.87 (1.38, 2.53)***	1.41 (1.15, 1.74)***
	Obesity class II	2.56 (2.01, 3.27)***	3.46 (2.71, 4.43)***	2.28 (1.67, 3.12)***	2.23 (1.75, 2.84)***
Hypertension	No	1 (reference)	1 (reference)	1 (reference)	1 (reference)
	Yes	1.19 (1.01, 1.41)*	1.12 (0.95, 1.33)	1.27 (1.02, 1.58)*	1.07 (0.90, 1.26)
Diabetes	No	1 (reference)	1 (reference)	1 (reference)	1 (reference)
	Yes	1.12 (0.86, 1.46)	2.16 (1.69, 2.76)***	0.75 (0.55, 1.03)	1.52 (1.19, 1.95)***
Cardiovascular disease	No	1 (reference)	1 (reference)	1 (reference)	1 (reference)
	Yes	0.98 (0.79, 1.21)	0.94 (0.78, 1.13)	1.08 (0.83, 1.40)	0.95 (0.78, 1.15)
Physical activity	Low	1 (reference)	1 (reference)	1 (reference)	1 (reference)
	Moderate	0.90 (0.72, 1.13)	0.92 (0.74, 1.15)	1.02 (0.77, 1.36)	1.00 (0.81, 1.23)
	High	0.73 (0.60, 0.89)**	0.79 (0.66, 0.95)*	0.76 (0.60, 0.96)*	0.87 (0.74, 1.02)
Sedentary	No	1 (reference)	1 (reference)	1 (reference)	1 (reference)
	Yes	1.17 (0.92, 1.49)	1.05 (0.84, 1.32)	1.32 (0.98, 1.77)	1.01 (0.81, 1.27)
Fruit/vegetable intake	≥ 5 servings	1 (reference)	1 (reference)	1 (reference)	1 (reference)
	< 5 servings	1.09 (0.86, 1.37)	0.94 (0.75, 1.16)	1.31 (0.98, 1.74)	0.92 (0.75, 1.24)
Current tobacco use	No	1 (reference)	1 (reference)	1 (reference)	1 (reference)
	Yes	1.23 (1.00, 1.50)*	1.43 (1.18, 1.45)***	1.24 (0.96, 1.60)	1.27 (1.03, 1.56)*
Passive smoking	No	1 (reference)	1 (reference)	1 (reference)	1 (reference)
	Yes	0.92 (0.78, 1.09)	0.92 (0.78, 1.73)	1.05 (0.84, 1.30)	1.07 (0.90, 1.27)
Heavy episodic drinking	No	1 (reference)	1 (reference)	1 (reference)	1 (reference)
	Yes	1.07 (0.89, 1.28)	1.32 (1.11, 1.57)**	0.89 (0.70, 1.12)	0.94 (0.76, 1.14)

*AOR* adjusted odds ratio.

***p < 0.001; **p < 0.01; *p < 0.05.

### Associations with dyslipidaemia awareness and treatment

In the adjusted logistic regression analysis, older age, higher education, urban residence, higher general body weight, hypertension, and cardiovascular disease were positively associated with dyslipidaemia awareness. Among those who were aware of their state of dyslipidaemia, belonging to the Khalkh ethnic group was positively associated and overweight or obesity class I was negatively associated with the treatment of dyslipidaemia (see Table 5).

**Table 5:** Associations with prevalence of dyslipidaemia awareness and treatment.

Variable	Subcategory	Awareness	Treatment
		AOR (95% CI)	AOR (95% CI)
Age (years)	15–39	1 (reference)	1 (reference)
	40–69	2.65 (1.35, 5.18)***	3.05 (0.86, 10.78)
Gender	Female	1 (reference)	1 (reference)
	Male	1.19 (0.56, 2.52)	1.71 (0.72, 4.07)
Education (in years)	0–9	1 (reference)	1 (reference)
	10–11	1.25 (0.87, 5.85)	0.51 (0.16, 1.60)
	≥ 12	2.84 (1.15, 7.00)*	0.88 (0.36, 2.15)
Ethnic group	Other	1 (reference)	1 (reference)
	Khalkh	0.66 (0.32, 1.37)	6.77 (1.84, 25.84)**
Residence	Rural	1 (reference)	1 (reference)
	Urban	2.82 (1.35, 5.89)**	1.20 (0.54, 2.69)
Body Mass Index	Normal	1 (reference)	1 (reference)
	Underweight	–	–
	Overweight	7.91 (1.94, 32.25)**	0.20 (0.04, 0.95)*
	Obesity class I	9.53 (2.90, 31.28)***	0.17 (0.05, 0.58)**
	Obesity class II	10.71 (3.09, 37.18)***	0.32 (0.09, 1.16)
Hypertension	No	1 (reference)	1 (reference)
	Yes	2.00 (1.05, 3.80)*	1.65 (0.67, 4.07)
Diabetes	No	1 (reference)	1 (reference)
	Yes	1.78 (0.78, 4.02)	1.37 (0.58, 3.24)
Cardiovascular disease	No	1 (reference)	1 (reference)
	Yes	2.23 (1.10, 4.51)*	0.78 (0.36, 1.71)
Physical activity	Low	1 (reference)	1 (reference)
	Moderate	0.91 (0.38, 2.20)	0.93 (0.38, 2.29)
	High	1.10 (0.52, 2.31)	0.57 (0.22, 1.50)
Sedentary	No	1 (reference)	1 (reference)
	Yes	1.09 (0.45, 2.62)	1.43 (0.64, 3.18)
Fruit/vegetable intake	≥ 5 servings	1 (reference)	1 (reference)
	< 5 servings	0.52 (0.25, 1.07)	1.57 (0.74, 3.32)
Current tobacco use	No	1 (reference)	1 (reference)
	Yes	0.44 (0.18, 1.07)	1.94 (0.80, 4.70)
Passive smoking	No	1 (reference)	1 (reference)
	Yes	1.68 (0.94, 3.01)	0.90 (0.40, 2.00)
Heavy episodic drinking	No	1 (reference)	1 (reference)
	Yes	1.16 (0.54, 2.45)	1.11 (0.42, 2.94)

*AOR* adjusted odds ratio.

***p < 0.001; **p < 0.01; *p < 0.05.

## Discussion

The study presents novel national data on the prevalence and distribution of dyslipidaemia in people 15 years and older in Mongolia in 2019. The proportion of dyslipidaemia in Mongolia (58.6%) was higher than in China (> 18 years, 34.0%)^6^, lower than in Northern Ethiopia (≥ 20 years, 66.7%)^2^, lower than in Turkey (≥ 20 years, ≥ 79%)^8^, and Pakistan (≥ 20 years, 96%)^9^. The prevalence of high TC (26.9%) in this study was higher than in the 35 LMIC study (≥ 15 years, 7.1%)^5^, lower than in Northern Ethiopia (≥ 20 years, 30.8%)^2^, in Jordan (≥ 25 years, 44.3%)^7^, in Turkey (≥ 20 years, 43%) and Pakistan (≥ 20 years, 39.3%)^9^. Some of these prevalence differences may be attributed to the use of different cut-off points of lipid abnormality. The high proportion of dyslipidaemia in Mongolia may be attributed to change in dietary behaviour, increased sedentary lifestyle, rapid urbanization, change in labour intensity of work and improved economic status^2,19,26,28^.

The most prevalent dyslipidaemia component was high TG (31.7%), followed by high TC (26.9%), high LDL-C (26.9%) and low HDL-C (14.6%). A similar rank order of prevalence of components of dyslipidaemia was found in Ethiopia, with low HDL-C having the lowest prevalence^2^, and in northeast China with the highest being high TG, followed by high TC^11^, while in national China the highest component was high low HDL-C, followed by high TG, high LDL-C and high TC^6^. The prevalence of high TG (31.7%) in this study was lower than in Northern Ethiopia (40.2%)^2^, in Turkey (35.7%)^8^, and Pakistan (48.9%)^9^. The prevalence of high LDL-C (26.9%) in this study was higher than in 35 LMIC (7.5%)^5^, lower than in northern Ethiopia (49.5%)^2^, Turkey (36.2%)^8^, and in Pakistan (39.7%)^9^. The prevalence of low HDL-C (14.6%) in this survey was lower than in Jordan (59.5%)^7^, in Turkey (41.5%)^8^, and Pakistan (> 80%)^9^.

Consistent with previous research^2,6,8,10–12^, older age, urban residence, obesity, hypertension and diabetes increased the odds of dyslipidaemia. Dyslipidaemia may increase with age because physical activity levels decrease leading to more fat accumulation^2^. Lifestyle changes, such as change in dietary and sedentary behaviour, may explain that with urbanization higher rates of dyslipidaemia were found^9,26^. The tendency to have a higher prevalence dyslipidaemia with higher levels of BMI can be attributed to “global metabolic effects of insulin resistance and an excess of visceral fat^27^.” A nutritional transition in Mongolia may have led to the found high prevalence of central obesity (56.7%) and high levels of body fat^28^, increasing the risk of dyslipidaemia.

Some studies in China^6,10^, found that male sex was positively associated with dyslipidaemia, while our study found a weak negative association between male sex and dyslipidaemia, mainly due to higher high TC and higher low HDL-C in men than women. We did not find a significant association between ethnicity and dyslipidaemia, contrary to some previous studies^10^. While some research^6^ showed a positive association between cardiovascular diseasen] and dyslipidaemia, we did not find this association. In unadjusted analyzes we found that increased physical activity was negatively associated with the prevalence of dyslipidaemia, which is consistent with some previous research^2,9^. Contrary to previous findings^2,10,13^, we did not find significant associations between tobacco use, passive smoking, alcohol use, and dyslipidaemia. Furthermore, we found regional differences in the prevalence and awareness of dyslipidaemia, such that prevalence and awareness was the lowest in the Western region (48.8%, and 3.3%, respectively) and among the highest in Ulaanbaatar (61.8%, and 8.5%, respectively). The role of geographic determinants of dyslipidaemia and awareness is important in designing effective intervention strategies^17^.

Factors associated with high TC in this study included older age, male sex, higher education, obesity, hypertension, physical inactivity, and current tobacco use. In previous studies^7,9,12,14,16^, older age, female sex, lower education, hypertension, diabetes, and obesity were associated with high TC. Factors associated with high TG in this study included older age, higher education, obesity, diabetes, physical inactivity, heavy episodic drinking, and current tobacco use. In previous studies^7,9,12,14^, male sex, higher education, hypertension, diabetes, obesity, and smoking or current tobacco use were associated with high TG. Factors associated with low HDL-C in this study included older age, male sex, higher education, obesity, hypertension, and physical inactivity. In previous studies, 7,14,15 decreased age, male sex, higher education, hypertension, diabetes, obesity, and smoking were associated with low HDL-C. Factors associated with high LDL-C in this study included lower age, female sex, being Khalkh, obesity, diabetes, and current tobacco use. In previous studies^7,14^, hypertension, diabetes, and obesity were associated with high LDL-C.

The prevalence of awareness of dyslipidaemia (6.2%), treatment (18.9%), and control (21.5%) were in terms of awareness and treatment lower than in the 35 LMIC study (31/36% and 29/33%, respectively) but higher in terms of control (7/19%)^5^. Awareness was also much lower than in China (31.0%) but treatment was similar to China (19.5%) and control was higher than in China (8.9%)^6^. In particular, the low awareness of dyslipidaemia emphasizes the need for opportunistic screening in Mongolia. Consistent with previous results^5,11,17^, older age, higher education, urban residence, obesity, hypertension, and cardiovascular disease were associated with increased awareness of dyslipidaemia in this study. The association between higher education, urban residence and higher awareness of dyslipidaemia may be explained by higher health literacy in urban educated Mongolians and better access to health care in urban settings^17^. The association between general obesity and awareness of dyslipidaemia may be attributed to lipids being part of weight management, yet overweight and obesity were negatively associated with dyslipidaemia treatment. Similar results were found for dyslipidaemia control in China, highlighting the greater difficulty of dyslipidaemia control among obese compared to people of normal weight^17^.

The strengths of the study include the use of a large nationally representative sample and standardized STEPS methodology and measures. Some variables were evaluated by self-report, which may have biased responses, and the cross-sectional design precludes causative conclusions between the evaluated variables. The sample was restricted to those with complete lipid measurements. The excluded participants were largely similar to those included in the analysis. We conducted additional analyses with imputed data, which provided similar results. Familial hyperlipidaemia and more detailed dietary behaviour were not assessed and should be included in future research. Another limitation was that the 2019 Mongolia STEPS survey only included people aged 15–69 years, while the pattern of dyslipidaemia in people aged ≥ 70 years is unknown.

## Conclusion

Six in ten Mongolians 15 years and older had dyslipidaemia. Several factors, including sociodemographic and health factors, were identified for dyslipidaemia. The high prevalence of dyslipidaemia in Mongolia warrants enhanced public health interventions, including screening, better diagnose, treat, and control dyslipidaemia.

## Supplementary Information

Supplementary Table 1.

## Data Availability

The data source is publicly available at the World Health Organization NCD Microdata Repository (URL: https://extranet.who.int/ncdsmicrodata/index.php/catalog).
