# Supplementary material for: National high prevalence, and low awareness, treatment and control of dyslipidaemia among people aged 15–69 years in Mongolia in 2019
Source: Sci Rep. 2022 Jun 21;12:10478. doi: 10.1038/s41598-022-14729-2 (PMC9213429; doi:10.1038/s41598-022-14729-2)
Supplement: Supplementary file 1 — Supplementary Table 1. [file 41598_2022_14729_MOESM1_ESM.docx]

Supplementary Table 1: Multiple imputations associations with prevalence of dyslipidaemia

|  | Subcategory | COR (95% CI) | AOR (95% CI)^a^ |
| --- | --- | --- | --- |
| Age (years) | 15-39  40-69 | 1 (Reference)  1.37 (1.23-1.54)*** | 1 (Reference)  1.22 (1.04-1.43)* |
| Gender | Female  Male | 1 (Reference)  0.93 (0.82-1.06) | --- |
| Education (in years) | 0-9  10-11  ≥12 | 1 (Reference)  1.12 (0.96-1.31)  1.19 (1.02-1.39)* | 1 (Reference)  1.15 (0.98-1.35)  1.25 (1.08-1.44)** |
| Ethnic group | Other  Khalkh | 1 (Reference)  1.11 (0.93-1.32) | --- |
| Residence | Rural  Urban | 1 (Reference)  1.32 (1.13-1.54)*** | 1 (Reference)  1.26 (1.07-1.47)** |
| Body Mass Index | Normal  Underweight  Overweight  Obesity class I  Obesity class II | 1 (Reference)  0.99 (0.67-1.45)  1.41 (1.16-1.73)***  2.21 (1.88-2.59)***  3.83 (3.14-4.68)*** | 1 (Reference)  0.93 (0.63-1.39)  1.35 (1.09-1.66)**  1.92 (1.63-2.27)***  3.11 (2.51-3.85)*** |
| Hypertension | No  Yes | 1 (Reference)  1.60 (1.41-1.81)*** | 1 (Reference)  1.28 (1.09-1.50)** |
| Diabetes | No  Yes | 1 (Reference)  2.25 (1.78-2.84)*** | 1 (Reference)  1.68 (1.31-2.17)*** |
| Cardiovascular disease | No  Yes | 1 (Reference)  0.99 (0.85-1.15) | --- |
| Physical activity | Low  Moderate  High | 1 (Reference)  0.85 (0.73-0.99)*  0.79 (0.69-0.90)*** | 1 (Reference)  0.95 (0.80-1.12)  0.91 (0.80-1.05) |
| Sedentary | No  Yes | 1 (Reference)  1.16 (0.99-1.36) | --- |
| Fruit/Vegetable intake | ≥5 servings  <5 servings | 1 (Reference)  0.94 (0.82-1.08) | --- |
| Current tobacco use | No  Yes | 1 (Reference)  0.95 (0.84-1.07) | --- |
| Passive smoking | No  Yes | 1 (Reference)  1.00 (0.90-1.12) | --- |
| Heavy episodic drinking | No  Yes | 1 (Reference)  1.32 (1.14-1.53)*** | 1 (Reference)  1.21 (1.02-1.44)* |

^a^all variables significant (*p*<0.05) in unadjusted analyses were included in the adjusted model; ****p*<0.001; ***p*<0.01; **p*<0.05; COR=Crude Odds Ratio; AOR=Adjusted Odds Ratio;
